# GEN3VA: aggregation and analysis of gene expression signatures from related studies

**DOI:** 10.1186/s12859-016-1321-1

**Published:** 2016-11-15

**Authors:** Gregory W. Gundersen, Kathleen M. Jagodnik, Holly Woodland, Nicholas F. Fernandez, Kevin Sani, Anders B. Dohlman, Peter Man-Un Ung, Caroline D. Monteiro, Avner Schlessinger, Avi Ma’ayan

**Affiliations:** 1 Department of Pharmacological Sciences, One Gustave L. Levy Place, Box 1603, New York, NY 10029 USA; 2 Mount Sinai Center for Bioinformatics, Icahn School of Medicine at Mount Sinai, One Gustave L. Levy Place, Box 1603, New York, NY 10029 USA; 3 Fluid Physics and Transport Processes Branch, NASA Glenn Research Center, 21000 Brookpark Rd, Cleveland, OH 44135 USA; 4 Center for Space Medicine, Baylor College of Medicine, 1 Baylor Plaza, Houston, TX 77030 USA; 5 Daylesford, The Fairway, Weybridge, Surrey, KT13 0RZ UK

**Keywords:** Systems Biology, Microarrays, Data mining, Interactive reports

## Abstract

**Background:**

Genome-wide gene expression profiling of mammalian cells is becoming a staple of many published biomedical and biological research studies. Such data is deposited into data repositories such as the Gene Expression Omnibus (GEO) for potential reuse. However, these repositories currently do not provide simple interfaces to systematically analyze collections of related studies.

**Results:**

Here we present GENE Expression and Enrichment Vector Analyzer (GEN3VA), a web-based system that enables the integrative analysis of aggregated collections of tagged gene expression signatures identified and extracted from GEO. Each tagged collection of signatures is presented in a report that consists of heatmaps of the differentially expressed genes; principal component analysis of all signatures; enrichment analysis with several gene set libraries across all signatures, which we term *enrichment vector analysis*; and global mapping of small molecules that are predicted to reverse or mimic each signature in the aggregate. We demonstrate how GEN3VA can be used to identify common molecular mechanisms of aging by analyzing tagged signatures from 244 studies that compared young vs. old tissues in mammalian systems. In a second case study, we collected 86 signatures from treatment of human cells with dexamethasone, a glucocorticoid receptor (GR) agonist. Our analysis confirms consensus GR target genes and predicts potential drug mimickers.

**Conclusions:**

GEN3VA can be used to identify, aggregate, and analyze themed collections of gene expression signatures from diverse but related studies. Such integrative analyses can be used to address concerns about data reproducibility, confirm results across labs, and discover new collective knowledge by data reuse. GEN3VA is an open-source web-based system that is freely available at: http://amp.pharm.mssm.edu/gen3va.

## Background

Genome-wide mRNA expression profiling is a useful method to globally assess the state of intracellular gene-regulatory networks within mammalian cells. However, performing such studies by individual laboratories is expensive, and thus, in a typical study, only a few samples are analyzed, typically a group of 2–4 control samples that are compared to a group of 2–4 perturbation samples. Hence, each study generates one or few expression “signatures” that identify the difference between two or more conditions. When such studies are published in biomedical journals, it is required that the authors deposit their data into authorized repositories such as the Gene Expression Omnibus (GEO) [1] or ArrayExpress [2]. The purpose of this requirement is to enable others to reproduce the results and to reanalyze the data for further biological discovery. As of the middle of 2016, in GEO there are ~70,000 data series from published studies, where more than half of those are from mammalian cells. This large collection of datasets from GEO offers the opportunity to compare similar studies for consistency and further biological discovery. The concept of identifying, aggregating, reprocessing, and reanalyzing studies from GEO for the development of consensus “signatures” has been attempted previously. For example, to develop Gene Expression MetaSignatures (GEMS), McKenna and coworkers aggregated gene expression signatures from many studies that perturbed the estrogen receptor in MCF7 cells [3]. Their analysis was able to confirm known genes and discover novel genes regulated by 17beta-estradiol in MCF7 cells. Another example is the identification of resistance pathways in lung cancer by reprocessing a collection of gene expression data from a diverse set of lung cancer tumors [4]. The GEO database is well-structured, open, and free. It provides access through an application programming interface (API), and this enables the development of tools that “sit on top” of the GEO repository, potentially improving access and post-processing capabilities. For example, GEOMetaDB [5] was developed to enable improved search through reorganization of all the GEO metadata. To search for matching datasets, ExpressionBlast was developed [6]. ExpressionBlast is a search engine that matches single input signatures across all samples and species, processed from GEO automatically. Another tool, GEOquery [6], provides easier means to access and reprocess studies. However, on their own, GEOquery or ExpressionBlast are not sufficient to obtain global views of many related aggregated signatures that follow a specific theme. Consequently, we recently developed GEO2Enrichr [7], a web browser extension that enables novice users to extract signatures from GEO, add metadata to existing GEO entries, and reanalyze gene expression data from the published studies by piping the signatures into downstream analysis with tools such as Enrichr [8], principal angle enrichment analysis (PAEA) [9], and L1000CDS2 [10].

Here we present a new web-based software application called GENE Enrichment and Expression Vector Analyzer (GEN3VA). GEN3VA provides multi-level analysis of sets of related gene expression signatures extracted from GEO. Each set of gene signatures is processed into interactive reports. These reports provide a single-page summary with several types of interactive visualizations such as a 3D principal component analysis (PCA) scatterplot, and several heatmaps specifying gene signatures as the column labels, with the row labels corresponding to genes, enrichment terms, or small molecule compounds. Reports retain the original information from the gene signatures in the collection while allowing users to interrogate the signatures for multiple views. For example, users can create custom reports from subsets of gene expression signatures of their choice. We demonstrate the usefulness of GEN3VA by identifying common molecular mechanisms of aging. To achieve this, we analyze tagged signatures from 244 studies that compared young vs. old tissues from mammalian organisms. In a second case study, a collection of 86 signatures was created from studies in which mammalian cells were treated with dexamethasone, a glucocorticoid (GR) agonist. Our analysis of this collection of signatures confirmes consensus of GR target genes and predicts potential drug mimickers.

## Implementation

### Differential expression data processing

Most of the gene expression signatures contained in GEN3VA were extracted using GEO2Enrichr, a browser extension that enables the labeling and extracting of signatures directly from GEO pages [7]. To identify differentially expressed genes, GEO2Enrichr implements the characteristic direction (CD), a multivariate method that outperforms limma and SAM in several benchmarks [11]. The CD method uses the covariance matrix of the gene expression, and linear discriminant analysis (LDA), to first identify a hyperplane that maximally separates the control vs. the perturbation samples in N-dimensional gene expression space. Then differentially expressed genes are scored and ranked based on their alignment to the normal of this hyperplane. Such an approach places less emphasis on the magnitude of change and more emphasis on the direction of change as it relates to other genes. In several benchmarks we previously demonstrated that the CD method ranks higher more genes that are expected to be differentially expressed. For example, computing differential expression after transcription factor perturbations in mammalian cells, with the CD method, we observed that the method ranks higher putative targets of the transcription factors determined independently by ChIP-seq profiling. Differential expression is determined by a *p*-value threshold of 0.01 after the Benjamini-Hochberg correction. If a collection of gene signatures is uploaded directly to GEN3VA through the application programming interface (API), these processing steps are expected to be already handled before submission. GEN3VA only supports processing of data from human, mouse or rat. Gene symbols are converted to their human orthologs using HomoloGene. Probes for the same gene are averaged. Converting probes to genes is done through the annotation files for each platform available from GEO’s platform pages.

### Integration with GEO2Enrichr

The GEO2Enrichr Chrome Extension is integrated with GEN3VA. After installing the GEO2Enrichr Chrome Extension, it is possible to select samples from studies in GEO, add metadata, and tag the signatures. After processing a tagged signature, the signature and metadata are automatically posted into the GEN3VA database, and can be accessed under the Collections tab. The Get Started tab provides a tutorial, and a search bar that allows users to find studies in GEO directly from the GEN3VA site. Above the search bar, users are notified whether they are using the Chrome browser and have the GEO2Enrichr extension installed.

### PCA scatterplots

To create the PCA scatterplots, the gene signatures in a collection are treated as column-vectors and concatenated horizontally. Each concatenation is an outer join. Genes with missing values are filled with zeros, indicating that they have no change in expression. The resulting matrix has gene signatures as column labels, genes as row labels, and scores from the differential expression method as cell values. PCA is performed using the scikit-learn library for machine learning in Python [12]. Plotting is achieved using HighCharts’ 3d scatter draggable: http://www.highcharts.com/demo/3d-scatter-draggable.

### Interactive heatmaps

The process for creating the matrices underlying each heatmap type is described in detail below. After each matrix is created, it is converted into a web-based, interactive heatmap using Clustergrammer [13].

#### Heatmap of differentially expressed genes

To create a heatmap of the differentially expressed genes, each gene signature in a collection is treated as a column vector. These column vectors are sorted and concatenated into a matrix in which missing values are filled with zeros, indicating no change in expression for those genes. The resulting matrix has gene signatures as the column labels, genes as the row labels, and scores from the differential expression analysis as the matrix data values.

#### Heatmaps of enrichment terms

To create the heatmaps of the enrichment terms, each gene signature is queried using the Enrichr API [14]. Each signature is transformed from a list of up or down genes from the differential expression analysis into a list of enriched terms and their scores from the enrichment analysis. To limit the size of the matrix for visualization, GEN3VA limits the returned list of enriched terms to the top 50 terms for each signature. These enrichment terms are treated as column vectors and concatenated horizontally. Each concatenation is an outer join. Missing values are filled with zeros, indicating no enrichment score. The resultant table has gene signatures as the column labels, enrichment terms as the row labels, and data values that are combined scores from the enrichment analysis computed by Enrichr. GEN3VA uses Enrichr’s “combined score” for prioritizing enriched terms. The combined score is a combination of a *p*-value computed by the hypergeometric test, and a z-score for the deviation of the term from its expected rank. It was demonstrated using several benchmarks that this method of enrichment outperforms the commonly used hypergeometric test [14]. GEN3VA performs the enrichment analysis twice for each signature, once for the up- and once for the down-regulated genes.

#### Heatmap of LINCS L1000 small molecule compounds

To create the heatmap of the Library of Integrated Network-based Cellular Signatures (LINCS) L1000 small molecule compounds, each gene signature is converted into a list of the top 50 small molecule compounds predicted to reverse or mimic the signature expression pattern using the web API developed for the L1000 Characteristic Direction Signature Search engine (L1000CDS^2^). L1000CDS^2^ uses processed data from the expression profiling studies of the LINCS L1000 dataset containing over 200,000 CD signatures for over 20,000 small molecule compounds [10]. L1000CDS^2^ uses the cosine distance to quantify the similarity between two signatures. The API returns the top 50 most similar or opposite small molecule compound-induced signatures based on signature similarity; GEN3VA performs this analysis twice to yield a list of compound-induced signatures that reverse and mimic the input gene signatures. For visualization purposes, GEN3VA translates the cosine distance score [0, 2] from L1000CDS^2^ to the cosine similarity score [1, −1]. In the heatmap, similar scores are rendered red, while compounds that reverse the signatures are rendered blue.

### Docking analysis

The atomic structure of the glucocorticoid receptor (GR) ligand-binding domain (LBD) bound to dexamethasone and the TIF2 coactivator protein (PDBID: 1M2Z [15]) was downloaded from the Protein Data Bank [16]. The GR LBD and the small molecules dexamethasone, ketorolac, and thalidomide were prepared for docking using Maestro v10.5 with OPLS3 force field. Ketorolac and thalidomide, two enantiomers each, as well as dexamethasone, were docked against GR, using Glide with the standard precision mode. The docking results were analyzed using the visualization program PyMOL [17].

### Web development technologies

The GEN3VA web application has two components: a back-end web server and a front-end user interface. The back-end is written in Python 2.7, and uses the Flask web framework. It runs on the Apache Server. The front-end is built using Jinja2, a web template system for Python. JavaScript and Cascading Style Sheets are used for scripting and styling, respectively. jQuery is used for browser API normalization; and Bootstrap is used for standardized, mobile-friendly layouts and interface components such as buttons and form fields. The application and its dependencies are packaged, deployed, and run inside a Docker container on a Hewlett-Packard 144 cores computer cluster running a Linux operating system.

## Results

### Developing collections of gene expression signatures from GEO

To develop GEN3VA, we collected differentially expressed gene signatures from GEO [18] and tagged these signatures based on their shared themes. The tags are keywords used to aggregate signatures into collections. In total, as of November 2016, the GEN3VA database contains 21,716 total signatures, 64894 gene sets, 276 tags, and 181 reports. Many of the gene expression signatures in GEN3VA were collected by students who participated in two Massive Open Online Courses (MOOCs) on Coursera: Network Analysis in Systems Biology (NASB) [19] and Big Data Science with the BD2K-LINCS Data Coordination and Integration Center (DCIC) [20]. These students extracted these signatures as a part of a voluntary crowdsourcing project that was independent from the course.

### The GEN3VA user interface

**Fig. 1:**
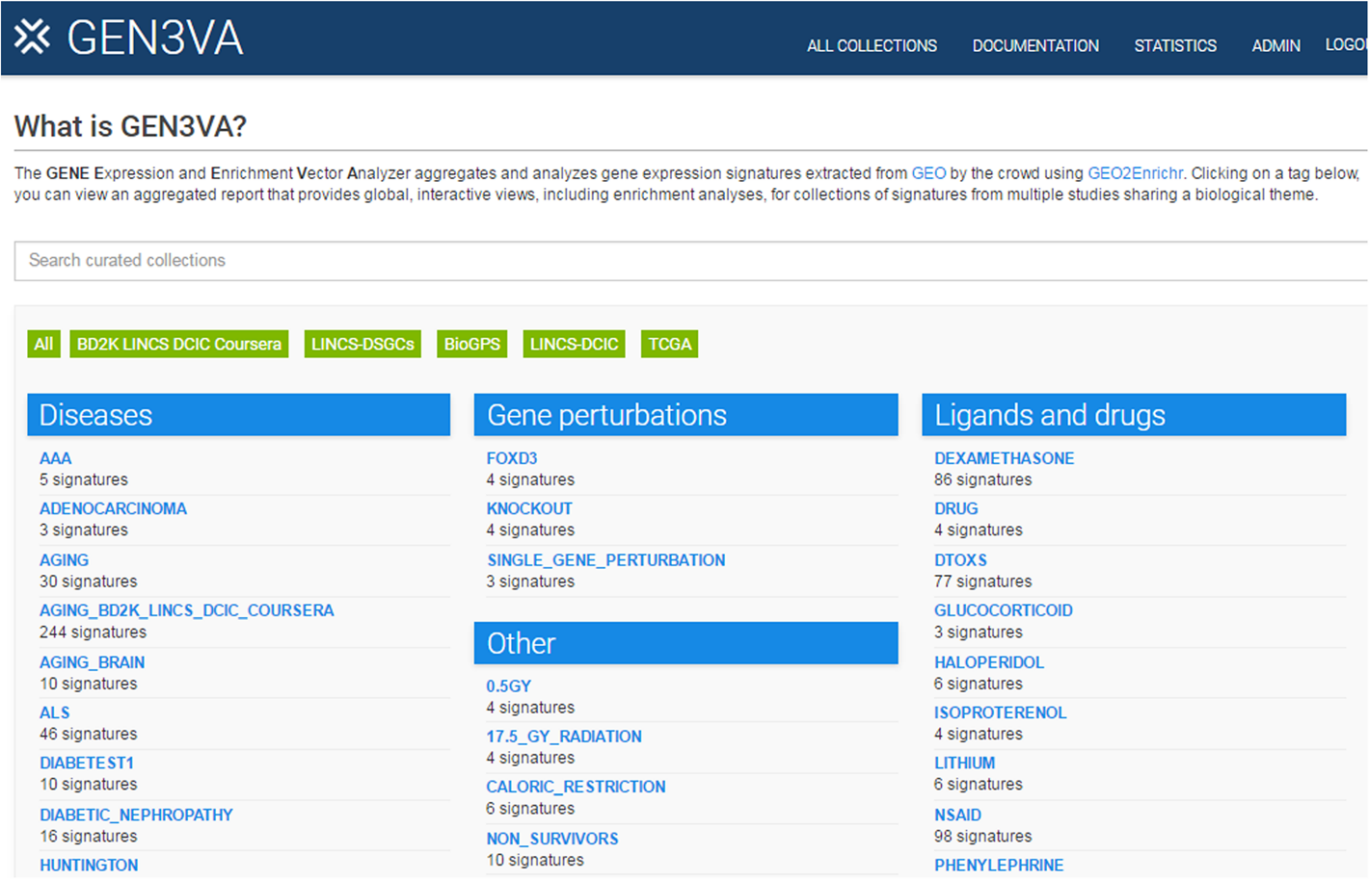
Screenshot from the GEN3VA landing page

**Fig. 2:**
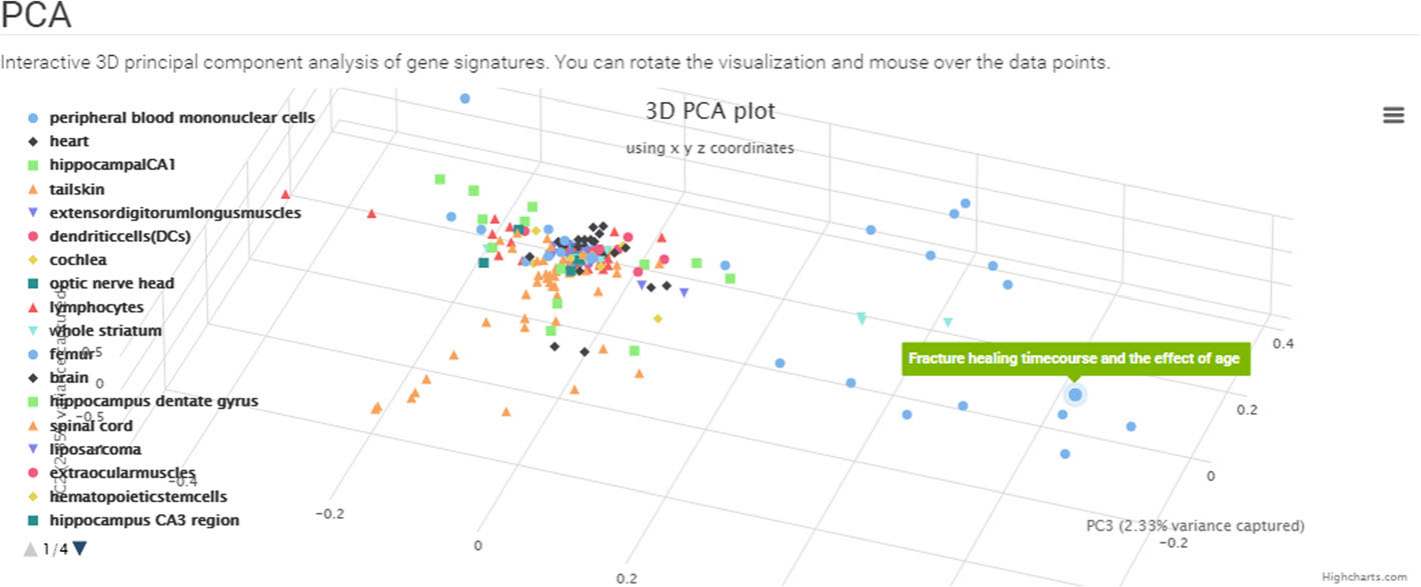
Screenshot from the 3D PCA analysis of the aging signature collection

The GEN3VA landing page contains curated collections of signatures that are grouped into five categories: (1) Diseases (e.g. Huntington’s or Parkinson’s); (2) Gene Perturbations (e.g. FOXD3); (3) Ligands and Drugs (e.g. tamoxifen or sunitinib); (4) Tissues and Cell Lines (e.g. MCF10A or fibroblast); and (5) Other (e.g. caloric restriction) (Fig. 1). A search bar enables users to filter the visible collections. The main menu contains a link to “All Collections”, a page with links to the full set of collections that currently exist in the GEN3VA database. Each collection has an associated report page with several types of interactive visualizations for interrogating the aggregated gene expression signatures. The interactive principal component analysis (PCA) shows the dimensionality-reduced distances between each signature represented as a point on the 3D interactive scatter plot (Fig. 2). A user can hover over a data point to see the metadata associated with each point. For some reports, points on the PCA plot are colored by their shared metadata across a subset of signatures.

**Fig. 3:**
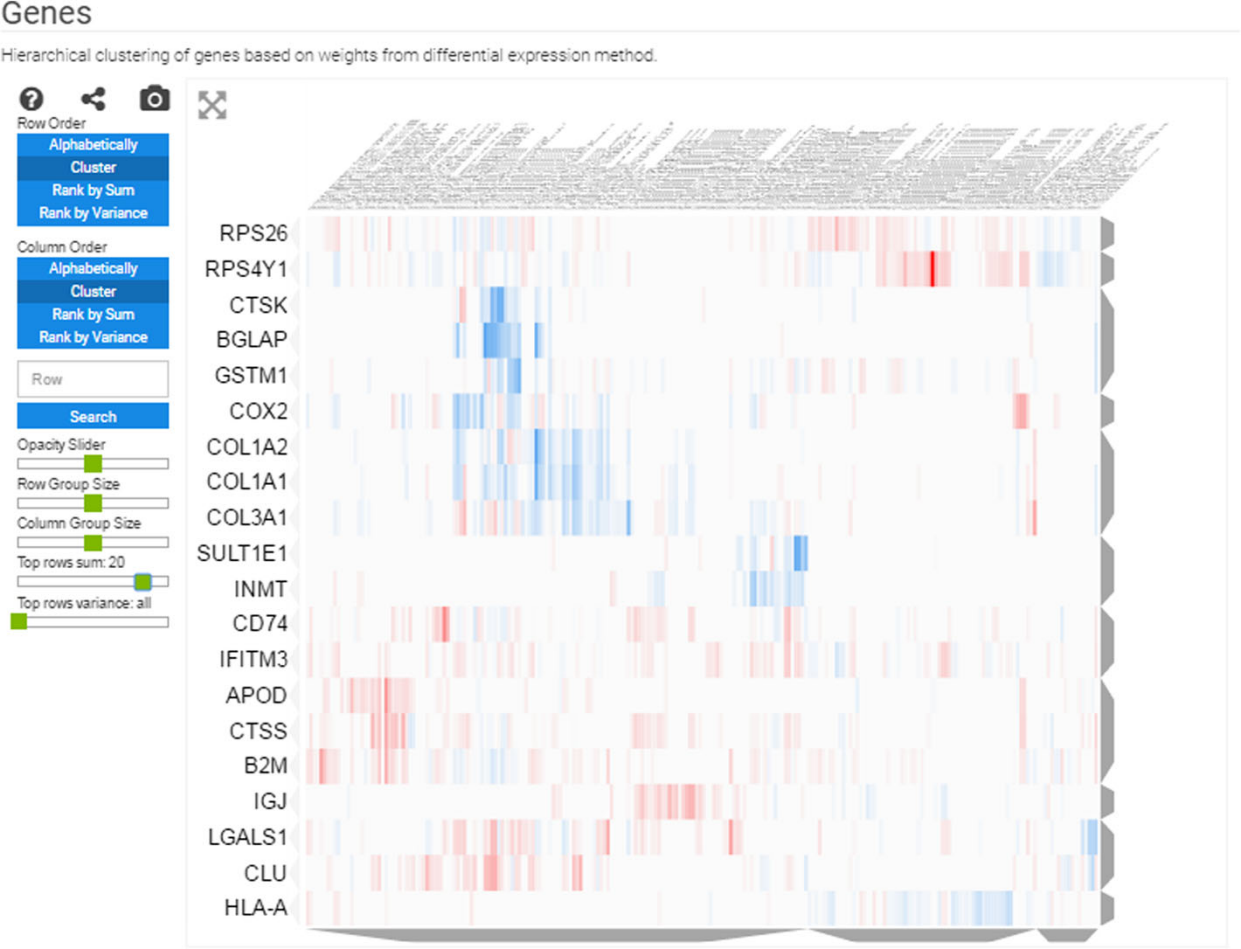
Screenshot from the genes heatmap of the aging signature collection showing the top 10 most up- and down-regulated genes across all studies

**Fig. 4:**
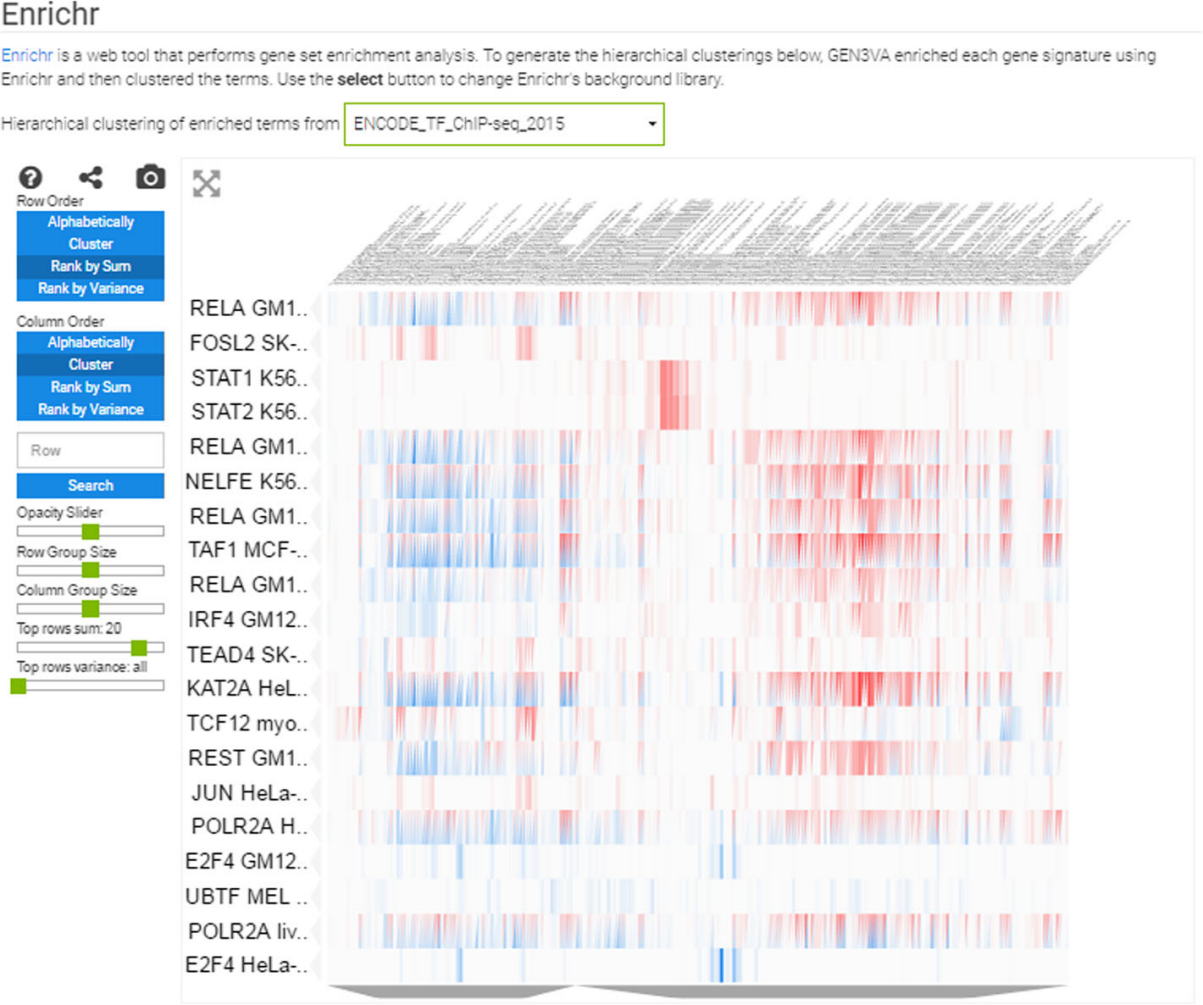
Screenshot from the enrichment analysis heatmap of the aging signature collection using the ENCODE library with a filter for the top 20 most consistently enriched terms

**Fig. 5:**
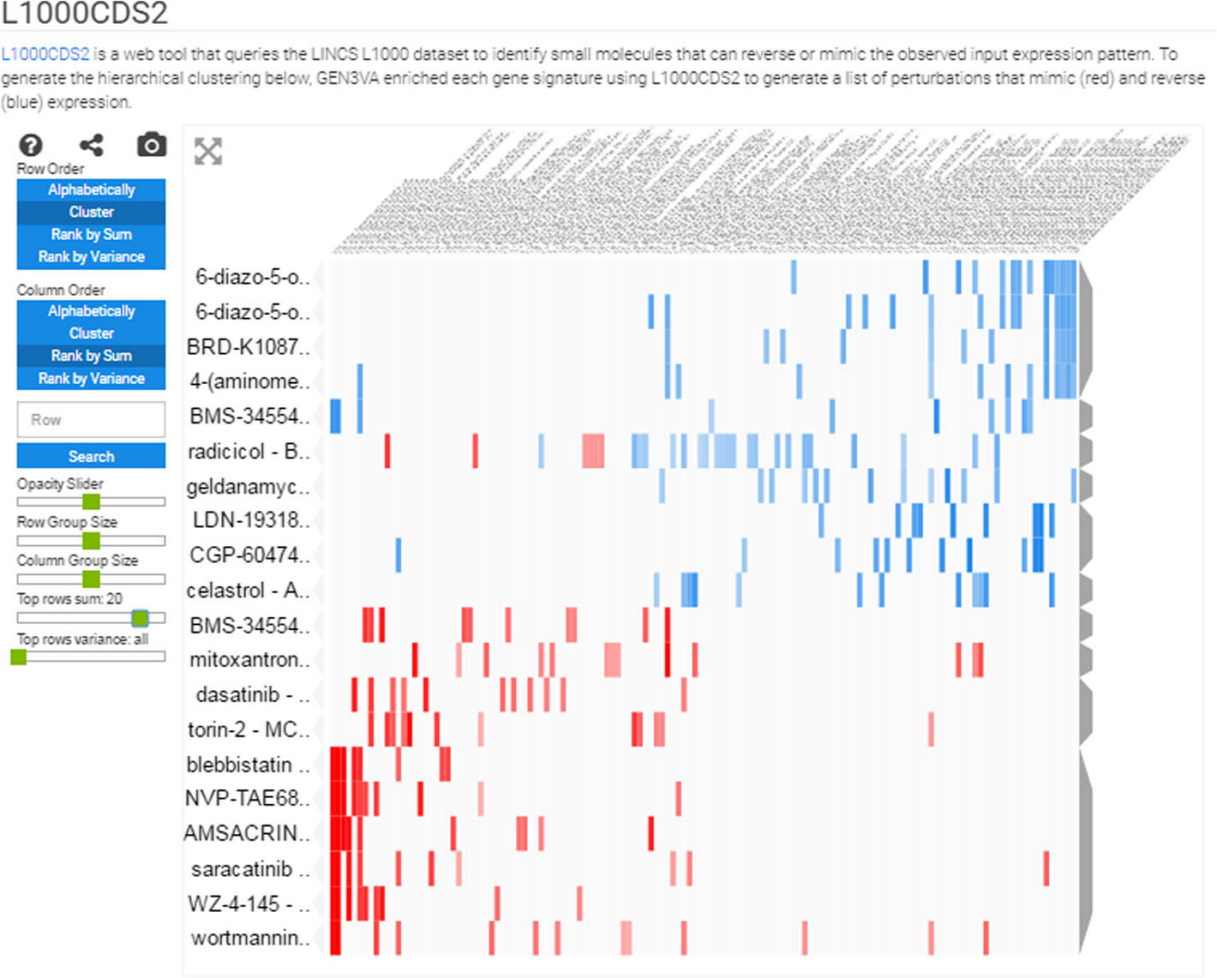
Screenshot from the L1000 drug-induced signatures enrichment analysis heatmap of the aging signature collection. *Blue spots* are reversers of the expression signatures, and *red spots* are mimickers. The filter is set to the overall top 20 most consistent enriched drugs

Reports also contain three types of heatmaps; these are interactive clustergrams with panning, zooming, filtering, searching, and reordering features. The column labels are the GEO signatures in a collection, whereas the rows, depending on the type of heatmap, are: (1) the differentially expressed genes filtered by the greatest sum of change as computed by the CD method across all signatures (Fig. 3); (2) the enrichment terms computed for each signature; these enriched terms are computed using the Enrichr API [14] (Fig. 4); or (3) the names of small molecule compounds that are predicted to reverse or mimic each gene expression signature by querying each signature against a subset of the LINCS L1000 dataset with the L1000CDS^2^ tool (Fig. 5). These three types of heatmaps offer different perspectives that enable better understanding of the themed gene expression collection. The heatmaps also intuitively visualize the level of consistency across signatures.

The most unique feature of GEN3VA is the enrichment vector analysis and visualization. Currently, GEN3VA supports enrichment term heatmaps for nine gene set libraries created from the Gene Ontology [21], the Kyoto Encyclopedia of Genes and Genomes (KEGG) pathways database [22], the Mouse Genome Informatics (MGI) Mammalian Phenotype Ontology (MPO) [23], the ENCyclopedia Of DNA Elements (ENCODE) project ChIP-seq data for mammalian transcription factors [24], the Epigonomics Roadmap for histone modifications associated with genes as determined by ChIP-seq [25], the ChIP-X Enrichment Analysis (ChEA) database [26], and a database of protein-protein interactions extracted manually from literature [27]; the final two libraries were constructed from single-gene perturbation studies from GEO (see Methods [28]). These libraries were chosen from a set of 90 libraries that exist in Enrichr [8]. We chose this subset of libraries since we have usage data indicating that those libraries are the most valuable and popular.

GEN3VA supports the creation of custom collections and reports. Custom reports are created by selecting a subset of gene signatures from an existing collection. Users can also choose a metadata field upon which to categorize the PCA and heatmap visualizations. This is useful to examine whether a metadata field, for example, a specific cell line or a tissue, is associated with a cluster in the heatmaps, or contributes to the agreement between signatures. In addition, users can upload and tag an entire collection of gene expression signatures using either the GEN3VA API or through an upload form.

### The case study of aging

To demonstrate how the GEN3VA system can be used to discover new knowledge, we first describe a collection of gene expression signatures extracted from GEO in which young mammalian tissue was compared to old tissue. GEO has many studies that collected gene expression from young and old tissues. Such studies do not always focus on understanding the aging process, but the data collected from such studies can be used to accomplish this goal. Generating signatures from young vs. old tissues can shed light on common alterations in pathways that are associated with aging. Better understanding the mechanisms of aging at the molecular level can ultimately lead to the identification of small molecules that can potentially decelerate aging, and warn against using drugs that accelerate aging. It is important to note that the “young” samples were from completely mature but young adults, and not from tissue collected from individuals that are still undergoing development and maturation.

In total, we have collected 244 signatures from 64 studies. Of these, 70 signatures are from rat, 102 from mouse, and 72 signatures are from human subjects, covering 62 tissues and cell types. Using GEN3VA, we created a report for this collection of signatures. This report is available at: http://amp.pharm.mssm.edu/gen3va/report/approved/AGING_BD2K_LINCS_DCIC_COURSERA.

Principal component analysis of all these signatures shows that few studies have signatures that spread out and dominate the diversity of the collection. These include one study that compared fracture healing across ages, and another study that compared tissues of two strains of young and aged rats (Fig. 2). The heatmap of the differentially expressed genes identifies CTSS, CLU, IFITM3, B2M, and RPS4Y1 as the most commonly upregulated genes; and the genes CTSK, COL3A1, COL1A2, BGLAP, and COL1A1 as the most commonly downregulated genes in aged tissues when compared with matched young tissues. When filtering by variance, the top 10 genes are RPS4Y1, CST3, ATP6, KCNJ16, COX2, SLUT1E1, CTSK, COL1A2, BGLAP, and COL1A1 (Fig. 3). Many of these genes have previously been implicated in aging. For example, it has been reported that CLU is associated with hippocampal degeneration [29]; β2-microglobulin (B2M) is a pro-aging factor that was reported to also reduce neurogenesis [30]. Cathepsin K (CTSK) is a protease involved in bone remodeling, and bone gamma-carboxyglutamate protein (BGLAP) is a highly abundant secreted protein in bone. Hence, all of the most consistent top five downregulated genes are a part of the collagen system that is known to be altered in aging [31]. COL3A1, COL1A2, and COL1A1 have been marked to alter aging and mortality in knockout mice (MP0010768) based on the MGI-MPO [32]. COX2 is a target of many anti-inflammatory drugs, and although its activity and role in aging is controversial, it is clear that its involvement is central [33]. ATP6 is linked to mitochondrial function, which is also central to global aging processes [34]. These are only some genes that appeared in the top ten using two filters; other highly ranked genes should also be considered as candidates for further investigation.

The enrichment vector analysis identifies RELA as the most enriched regulator of the genes that increase in expression across all signatures. In fact, several ENCODE studies performed in different cell types list RELA as the top enrichment term when performing enrichment vector analysis with the ENCODE library (Fig. 4). RELA was previously reported to be a critical component of aging, and downregulation of this gene has led to extended life span in several organisms [35, 36]. The enrichment vector analysis with ENCODE also points to STAT1 and STAT2 as being significant. This pair of transcription factors has previously been identified to be involved in aging kidneys [37], and our analysis confirms a global pro-inflammatory mechanism. Finally, drugs that can potentially accelerate or attenuate aging include celastrol, which was reported to indirectly inhibit NFKB signaling [38, 39], and radicicol, which is potentially a HSP90 and topoisomerase inhibitor [40]. Both drugs are small-molecule natural compounds that could be tested for their effect on aging (Fig. 5). In summary, our analysis points to the known NFKB pathway involvement in aging, and suggests small molecules that can potentially attenuate this and other relevant pathways. Skepticism should be placed when critically examining these results since inflammation could be an outcome of aging independently of the aging process, and the small molecules celastrol and redicolol have been reported to bind to many different targets and have controversial effects on mammalian cells.

### The case study of dexamethasone

Dexamethasone is a compound that first saw clinical use in the 1950s. It is known to bind to the glucocorticoid receptor (GR) and acts as an anti-inflammatory steroid. Dexamethasone is an immunosuppressant, and it is used to treat a variety of diseases. Since it is a widely used clinical drug, it is well studied. As of the middle of 2016, a PubMed search for dexamethasone returns over 60,000 entries. We also noticed that there are numerous studies that profiled genome-wide gene expression before and after dexamethasone treatment applied to a variety of human and mouse cells. We then asked whether we can identify consistency among these studies, as well as potentially predict drug mimickers of dexamethasone. Such potential drug mimickers may be useful as alternatives to dexamethasone due to the severe side effects profile and differential response of individuals for this drug.

Hence, for the second GEN3VA case study, we first identified, tagged, and processed gene expression signatures from studies that profiled the global transcriptional changes observed after applying dexamethasone to different mammalian cells. All 86 dexamethasone signatures originated from studies that utilized cDNA microarrays and are deposited in GEO. Using GEN3VA, we generated a report that contains the automated analysis of this collection. This report is available at: http://amp.pharm.mssm.edu/gen3va/report/approved/Dexamethasone.

**Fig. 6:**
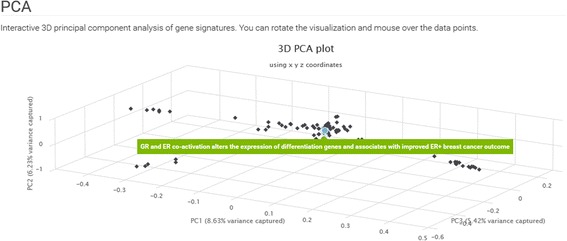
Screenshot from the 3D PCA analysis of the dexamethasone signature collection

**Fig. 7:**
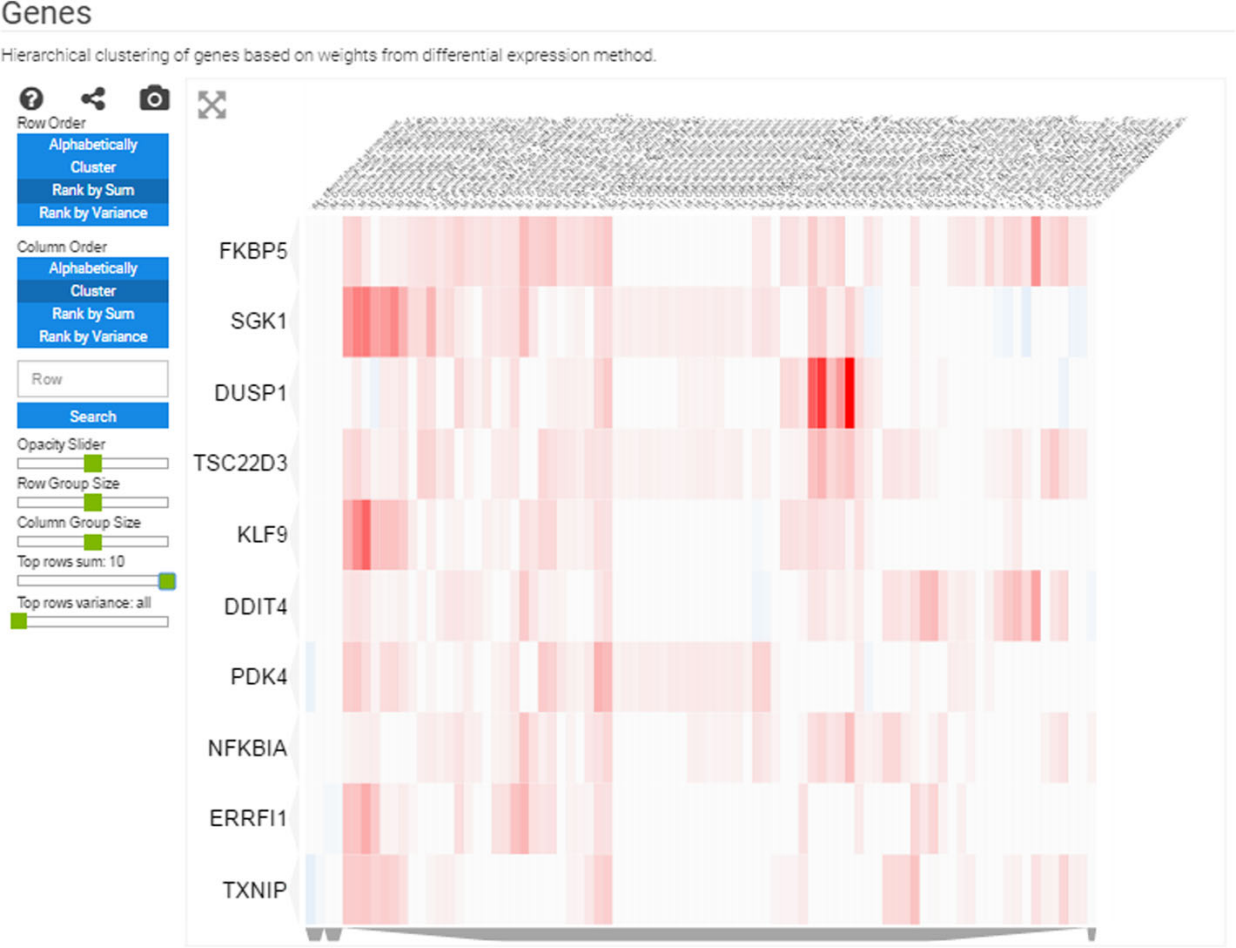
Screenshot from the genes heatmap of the dexamethasone signature collection showing the top 10 most up- and down-regulated genes across all studies

**Fig. 8:**
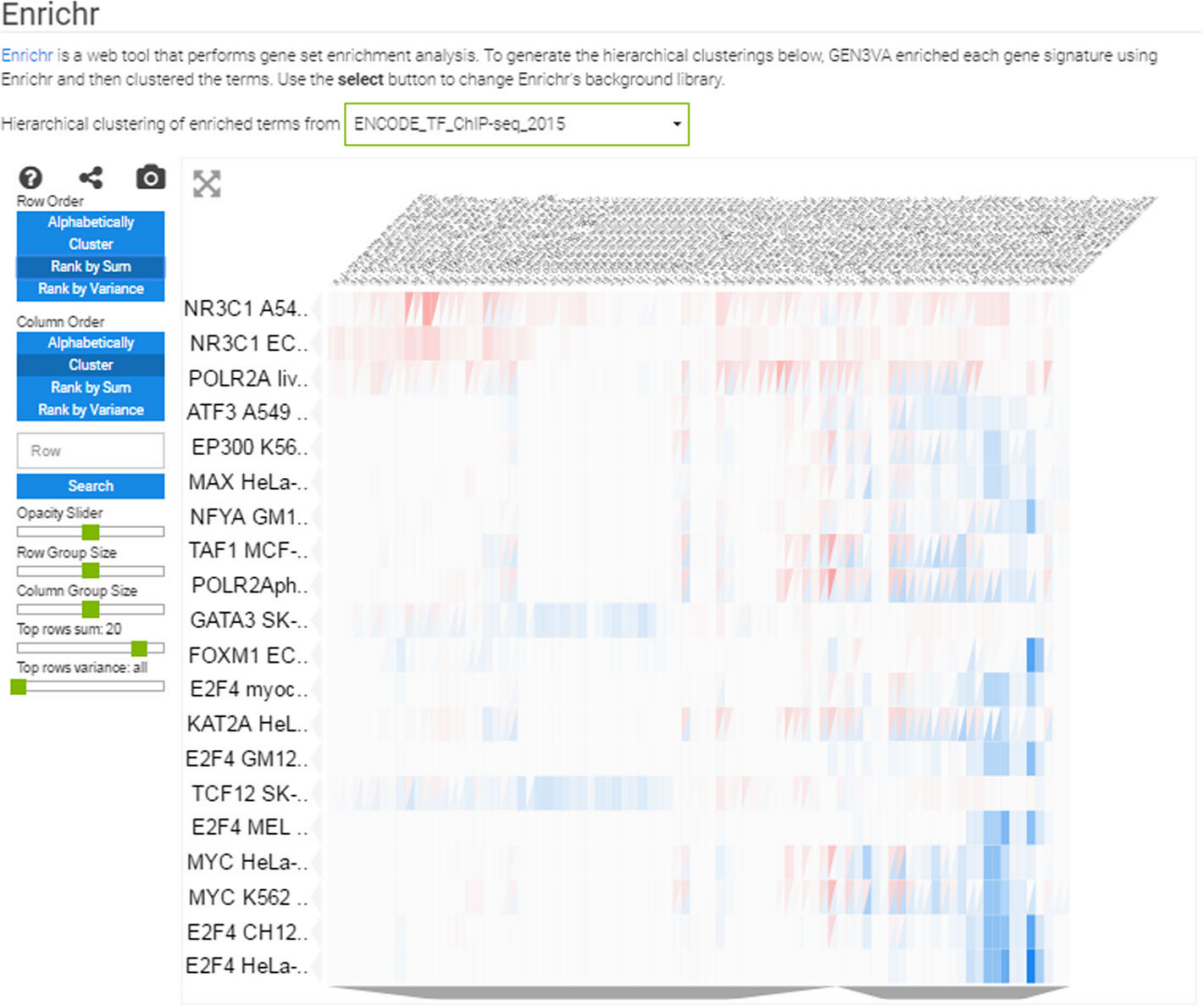
Screenshot from the enrichment analysis heatmap of the dexamethasone signature collection using the ENCODE library with a filter for the top 20 most consistently enriched terms

The PCA plot of all studies separates the studies into three clusters (Fig. 6). One of these clusters contains signatures from osteosarcoma cells, the second cluster contains only data from astrocytes, whereas the main cluster has all its signatures from other cell types. Among the top upregulated genes, NFKBIA stands out (Fig. 7). The NFKBIA gene encodes a protein that inhibits the NFKB pathway. Hence, the upregulation of the NFKBIA gene is likely a central mechanism for dexamethasone to exhibit its anti-inflammatory effects. To date, only one study has begun to explore this connection [41]. The imbalance of more upregulated genes compared with downregulated genes in the genes’ heatmap supports the role of dexamethasone as an activator of GR that induces the expression of its downstream targets. This is supported by the global enrichment analysis with the ENCODE gene set library, which shows that the most enriched transcription factors are GR (NR3C1) and polymerase 2 (POLR2A) (Fig. 8), suggesting increased transcription and transcriptional activity through GR.

**Fig. 9:**
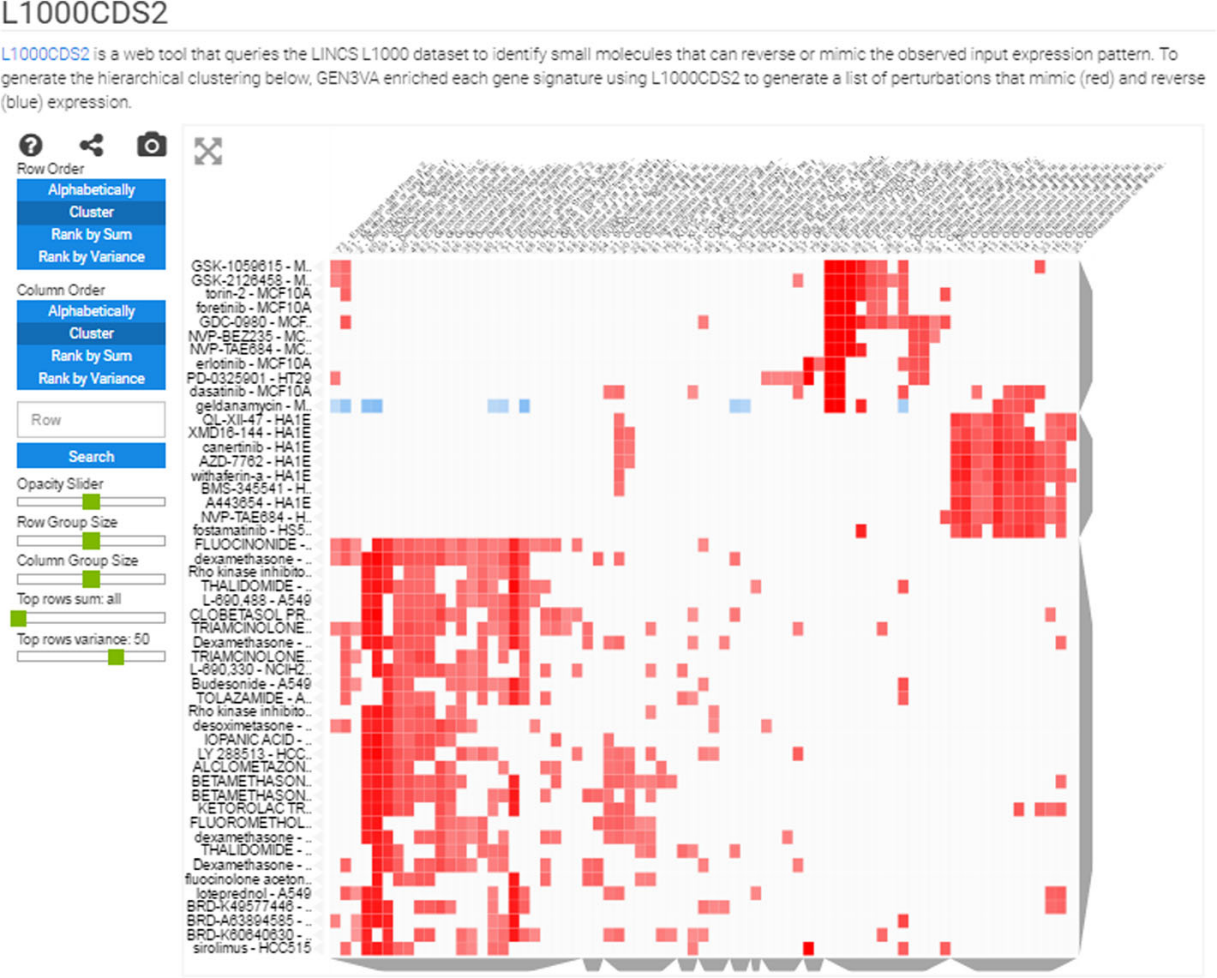
Screenshot from the L1000 drug-induced signatures enrichment analysis heatmap of the dexamethasone signature collection. *Blue spots* are reversers of the expression signatures, and *red spots* are mimickers. The filter is set to the overall top 50 most consistent enriched drugs

**Fig. 10:**
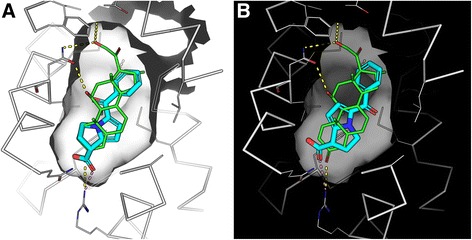
Docking of ketorolac and dexamethasone to the GR pocket. **a** zinc2279 (R)-ketorolac; **b** zinc11012 (S)-ketorolac. The white ribbon is the 1m2z structure, green stick is the ligand dexamethasone in crystal. Cyan stick is the ligand ketorolac

**Fig. 11:**
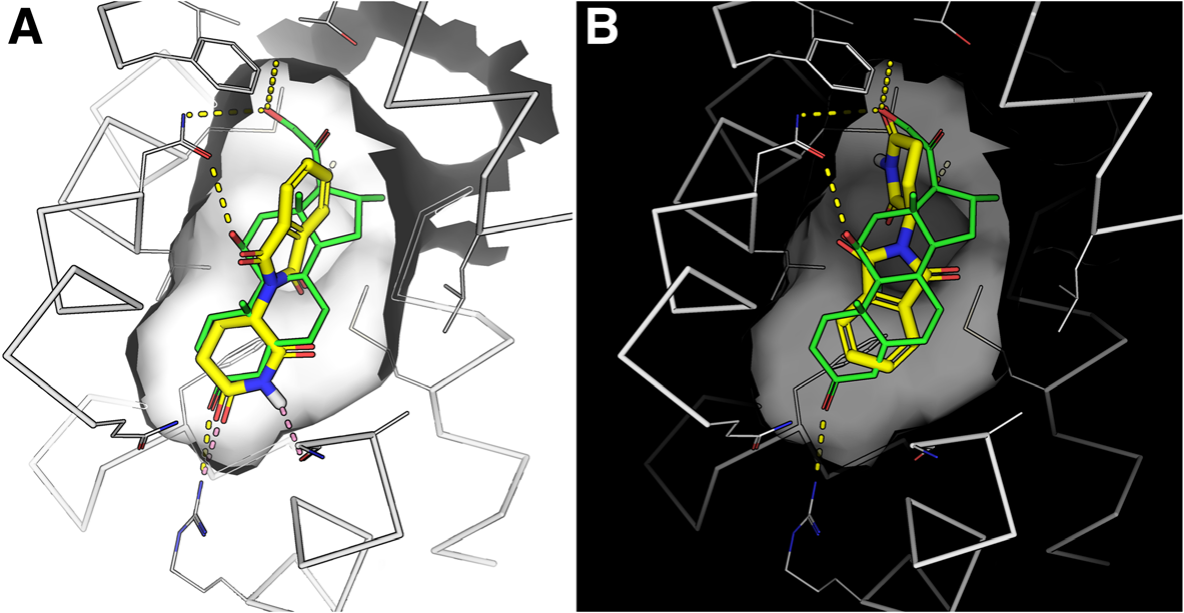
Docking of thalidomide and dexamethasone to the GR pocket. **a** (S)-thalidomide; **b** (R)-thalidomide

Finally, examining the predictions of small molecules that can mimic the effects of dexamethasone using the LINCS L1000 dataset, we observed a cluster that contains many entries that match LINCS L1000 dexamethasone signatures with the GEO signatures (Fig. 9). This cluster contains other drugs that are similar to dexamethasone, for example betamethasone, desoximetasone, and fluocinonide, which are all known glucocorticoids. However, the cluster also contains some surprises. For example, the drugs ketorolac and thalidomide have entries within this cluster. While these drugs are known to exert their anti-inflammatory effects through other molecular mechanisms, the close similarity in expression signature suggests that these drugs may also act directly on GR, perhaps when applied in high concentrations. To examine this possibility, we applied computational docking experiments to show that both drugs, ketorolac and thalidomide, can potentially fit in the same pocket where dexamethasone is known to bind (Figs. 10 and 11). Using Glide with all standard settings, glide can dock dexamethasone back almost in its crystal structure pose. There are two enantiomers for ketorolac: zinc2279 (R)-ketorolac, which has a slightly higher score than zinc11012 (S)-ketorolac. The marketed ketorolac is a racemic mixture. The carboxylic acid moiety of ketorolac interacts with the polar region of the pocket, namely the Gln570/Arg611, the same as the carbonyl moiety of dexamethasone from the structure 1m2z. Overall, ketorolac (−7.5; 255 mw) has much lower score than dexamethasone (−12.5; 392 mw). Similarly, the (S)-thalidomide (thal-s) docks better than the (R) enantiomer (thal-r) (Fig. 10). The Glide score for (S)-thalidomide is −9.3, whereas for (R) it is −8.5) (Fig. 11). Hence, the results overall are: (R)-ketoralac (−7.5; 255 mw) < (S)-thalidomide (−9.3; 258 mw) < dexamethasone (−12.5; 392 mw). Lower Glide score means predicted binding with more affinity. The fact that both thalidomide and ketorolac come in S and R forms can be used to direct experimental validation of physical binding by testing whether the different forms induce differential GR activity consistent with the computational docking. Finally, the cluster of dexamethasone mimickers also contains less-studied small molecules. Such chemicals should be considered as potential useful anti-inflammatory drugs and these include BRD-K49577446, BRD-A63894585, and BRD-K60640630.

## Conclusions

In summary, GEN3VA provides researchers with the opportunity to explore prior results from published studies by comparing and aggregating results from multiple related works performed by different labs around the world, using different assays and conditions. If common observations hold for the collection of studies, this implies that those results are robust and more likely reflect the real biology of the profiled cells. The interactive reports provide sers with the ability to explore their collections in the context of prior knowledge. The API enables users to upload entire collections of signatures, and this makes GEN3VA applicable to collections that extend beyond GEO.

Using GEN3VA, we demonstrate how the system can be used to confirm existing findings and discover new knowledge. We examined signatures from studies that compared young vs. old tissues to explore molecular mechanisms of aging. Most of the studies that we aggregated for the aging collection did not intend to study aging in particular or in general. Hence, this case study demonstrates that by aggregating collections of studies, expression data can be repurposed for answering new questions. The second case study includes a collection of signatures created from studies that profiled changes in expression due to dexamethasone treatment. Our analysis confirms that dexamethasone works through the transcription factor GR by activating genes that deactivate the NFKB signaling pathway. We saw that dexamethasone upregulates the expression of many genes while not reducing the expression of others. Our observation that many dexamethasone signatures from GEO match dexamethasone LINCS L1000 signatures suggests that dexamethasone works in a manner that is independent of cell type, and produces a robust response that can be detected across assay types, platforms, and organisms. While we detected that approximately one-third of the dexamethasone signatures from GEO confirmed dexamethasone activity across platforms, two-thirds of the studies did not. This does not mean that the quality of these studies is poor, but this observation should be further investigated for an explanation. On the other hand, while analyzing data from many studies using GEN3VA, users should be careful with disproportional contribution of signatures from the same study, or signatures from the same platform, or signatures from the same tissue/cells, or any other confounding factors that can inflate the importance of a gene, an enriched term, or a drug in the heatmaps across a diverse collection of signatures. For dealing with this, users can build their own custom reports by selecting a subset of signatures from each collection. Regardless of these considerations, in an era where data reproducibility is a growing concern, GEN3VA is providing an initial demonstration that order can emerge from the apparent disorder of disparate published studies in molecular systems biomedicine.

## Availability and requirements

Project name: GEN3VA.

Project home page: http://amp.pharm.mssm.edu/gen3va

Operating systems: Platform independent

Programming languages: Python, JavaScript, SQL.

License: GNU GPL v3

Source code: https://github.com/MaayanLab/gen3va

## Abbreviations

API: Application Programming Interface
BD2K: Big Data to Knowledge
BGLAP: Bone gamma-carboxyglutamate protein
CD: Characteristic direction
ChEA: ChIP-X Enrichment Analysis
CTSK: Cathepsin K
DCIC: Data Coordination and Integration Center
ENCODE: ENCyclopedia of DNA Elements
GEMS: Gene Expression Meta Signatures
GEN3VA: GENE Expression and Enrichment Vector Analyzer
GEO: Gene Expression Omnibus
GR: Glucocorticoid receptor
KEGG: Kyoto Encyclopedia of Genes and Genomes
L1000CDS2: L1000 Characteristic Direction Signature Search engine
LBD: Ligand Binding Domain
LINCS: Library of Integrated Network-based Cellular Signatures
MCF7: Michigan Cancer Foundation-7
MGI: Mouse Genome Informatics
MOOC: Massive Open Online Course
MPO: Mammalian Phenotype Ontology
NASB: Network Analysis in Systems Biology
NFKB: Nuclear Factor kappa-light-chain-enhancer of activated B cells
PAEA: Principal angle enrichment analysis
PCA: Principal component analysis
